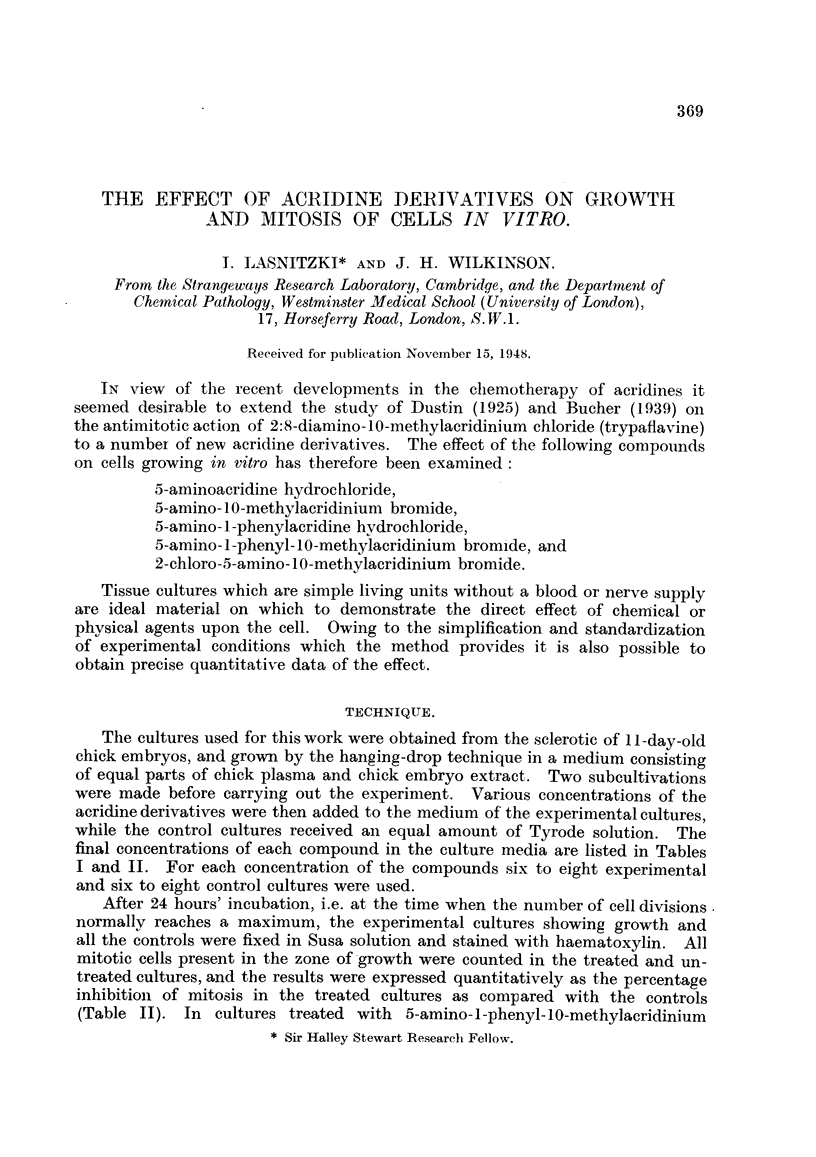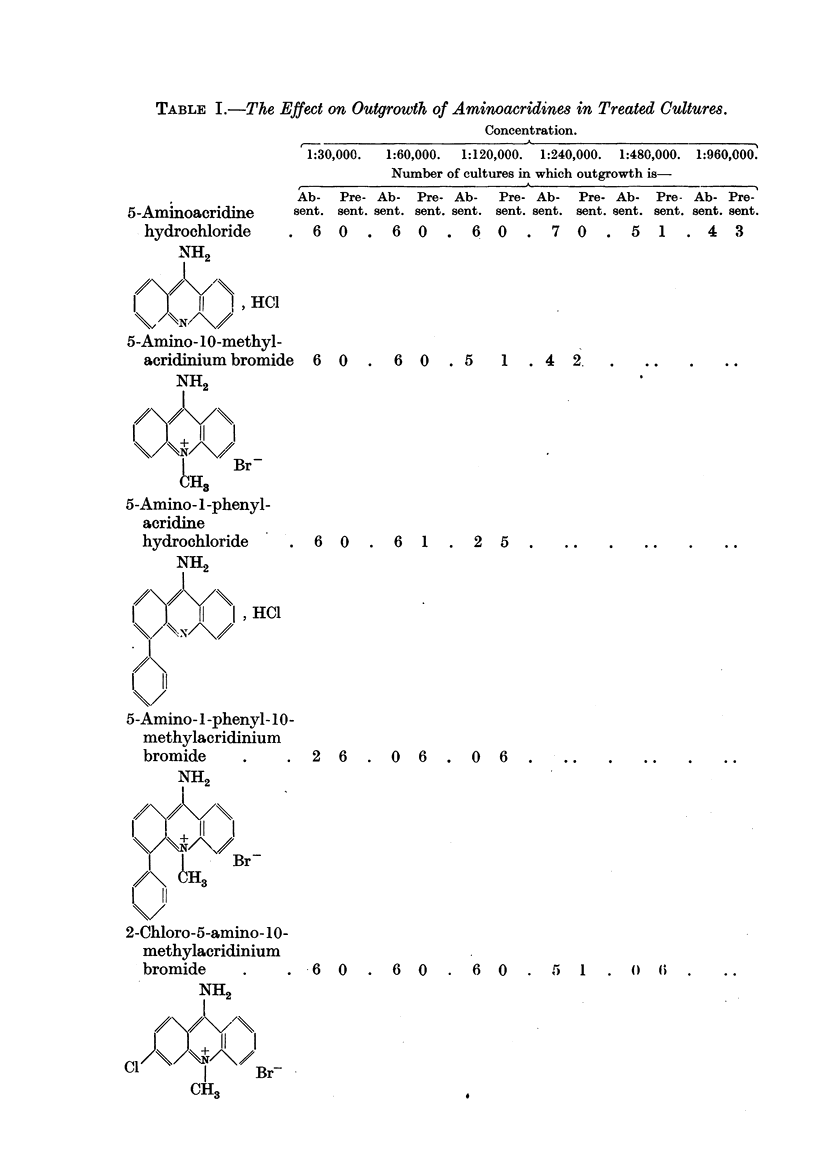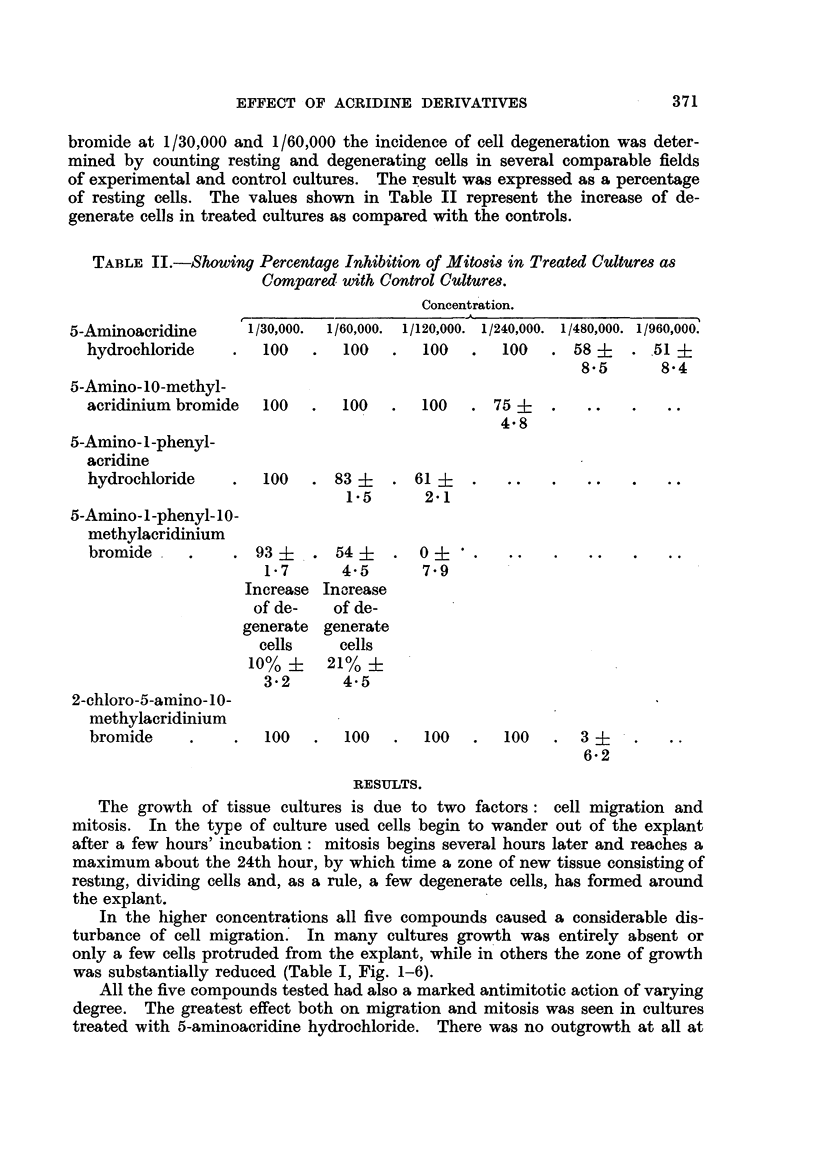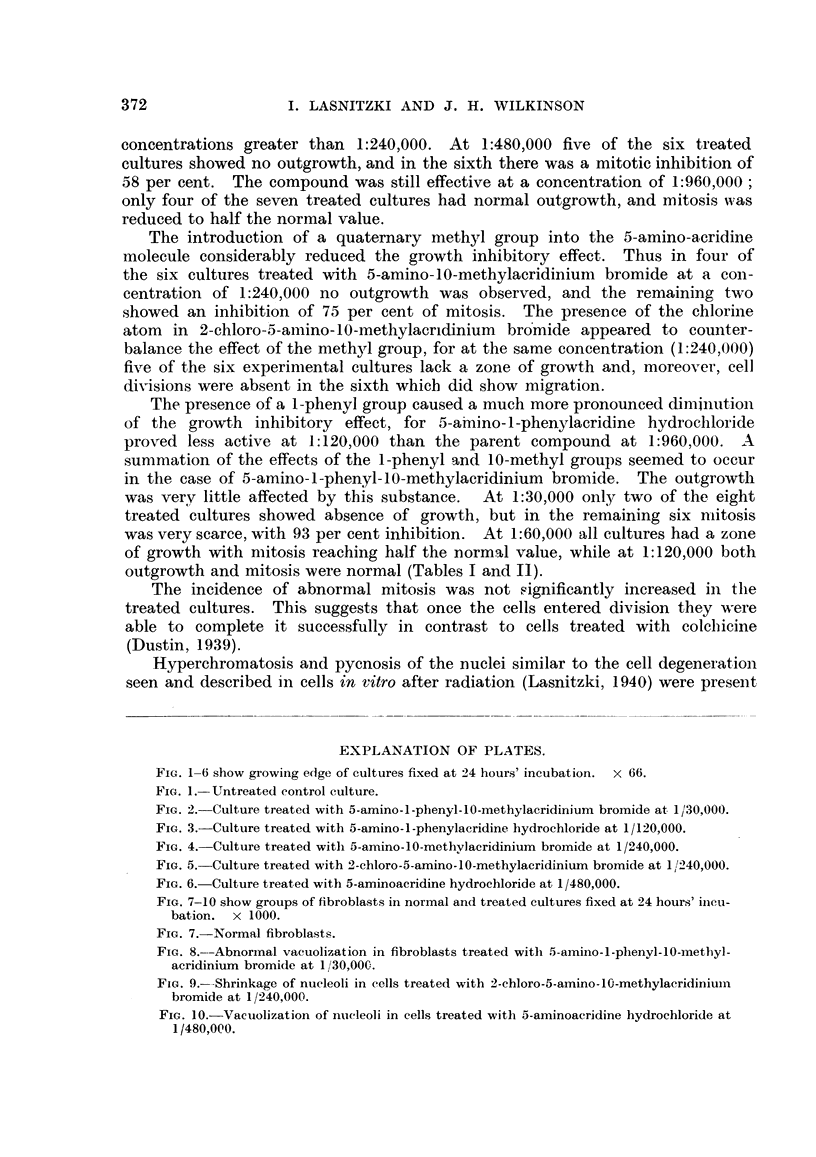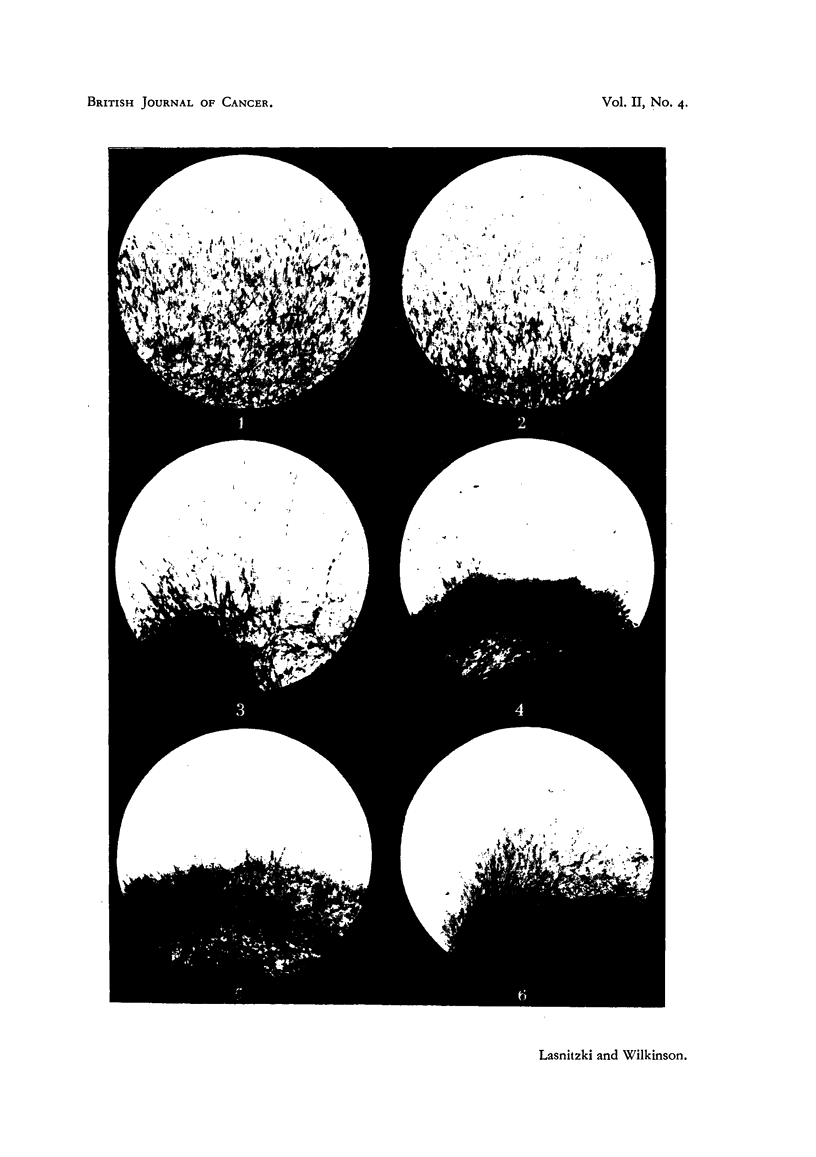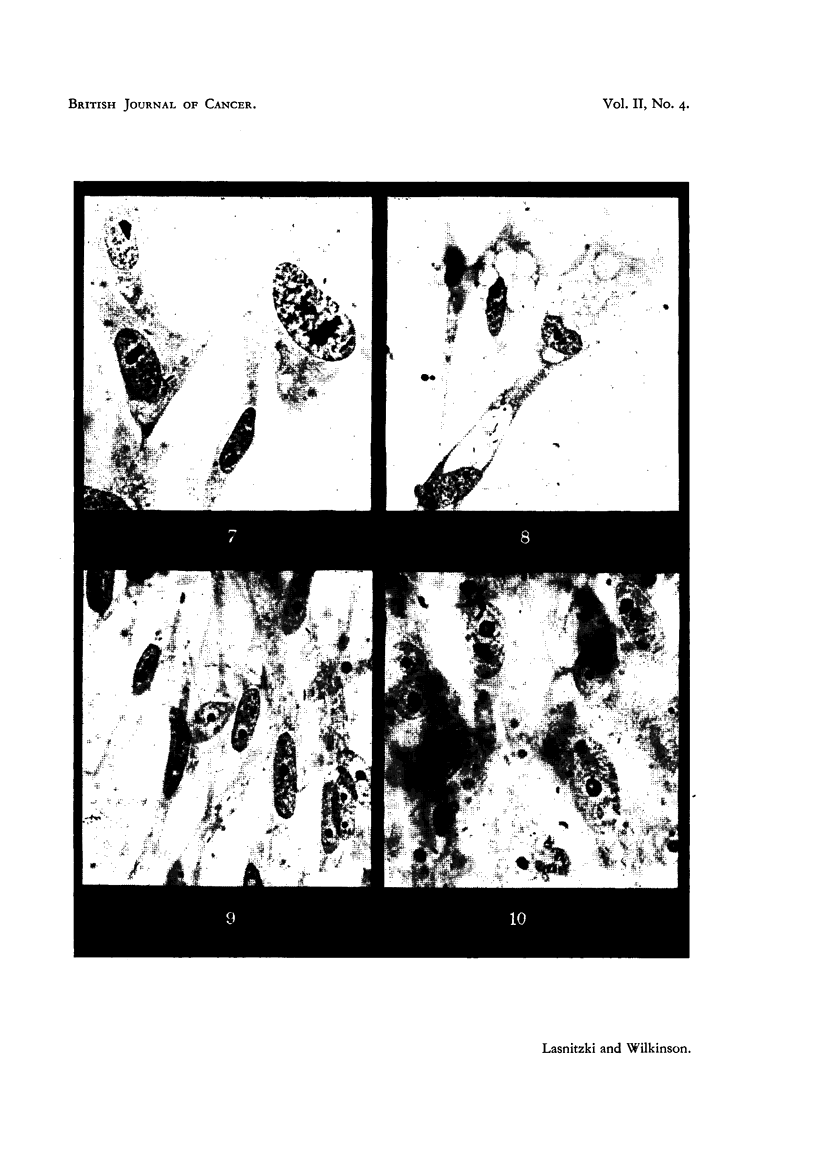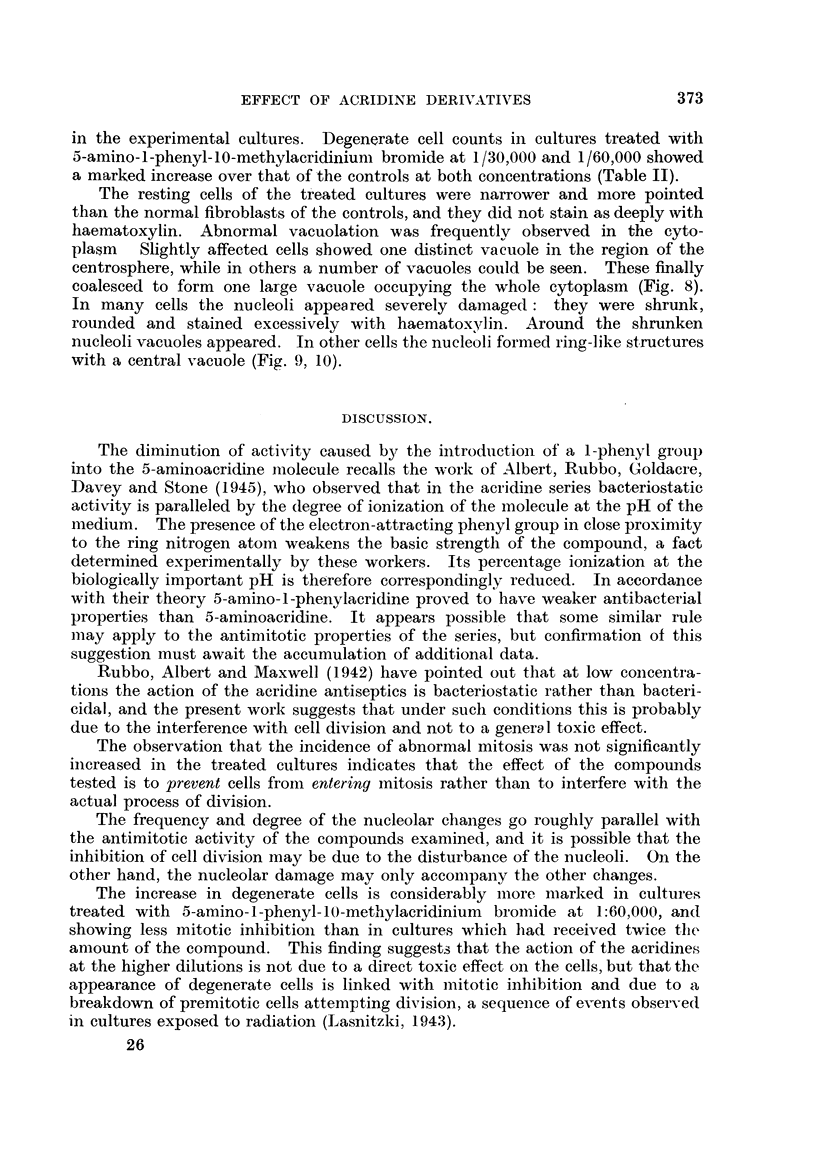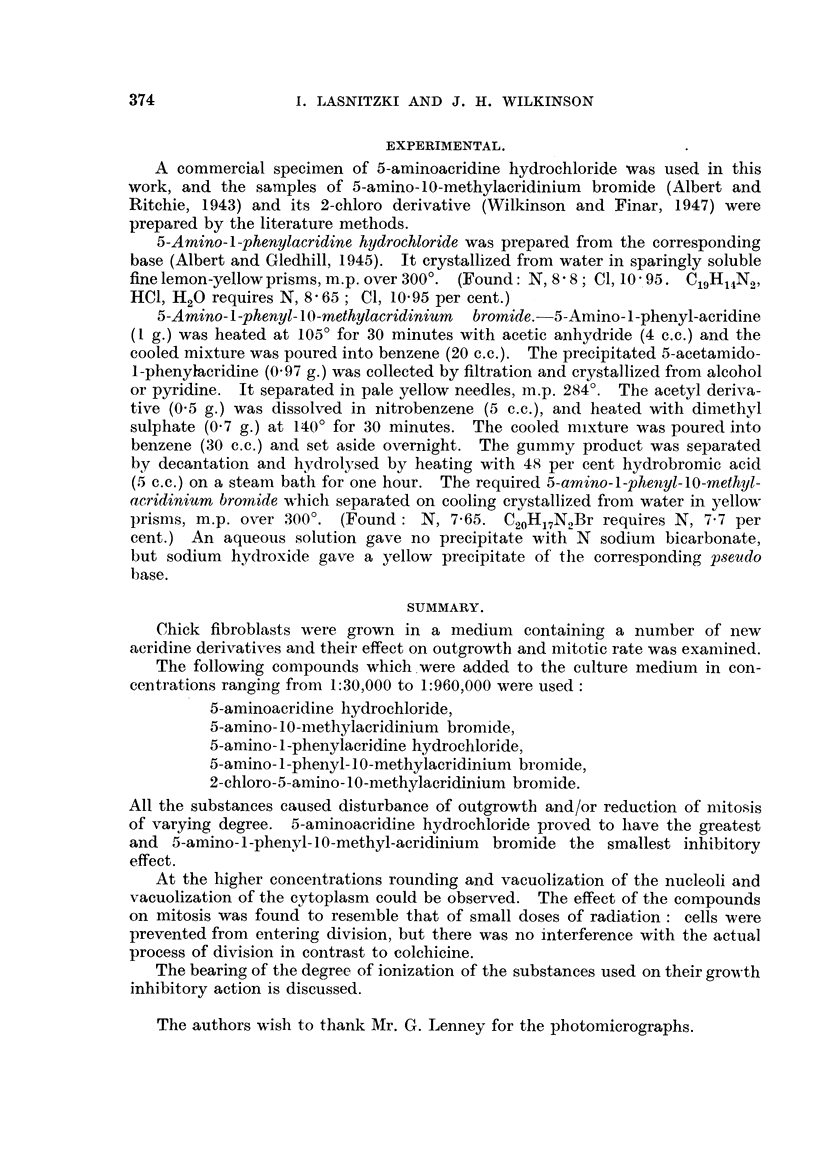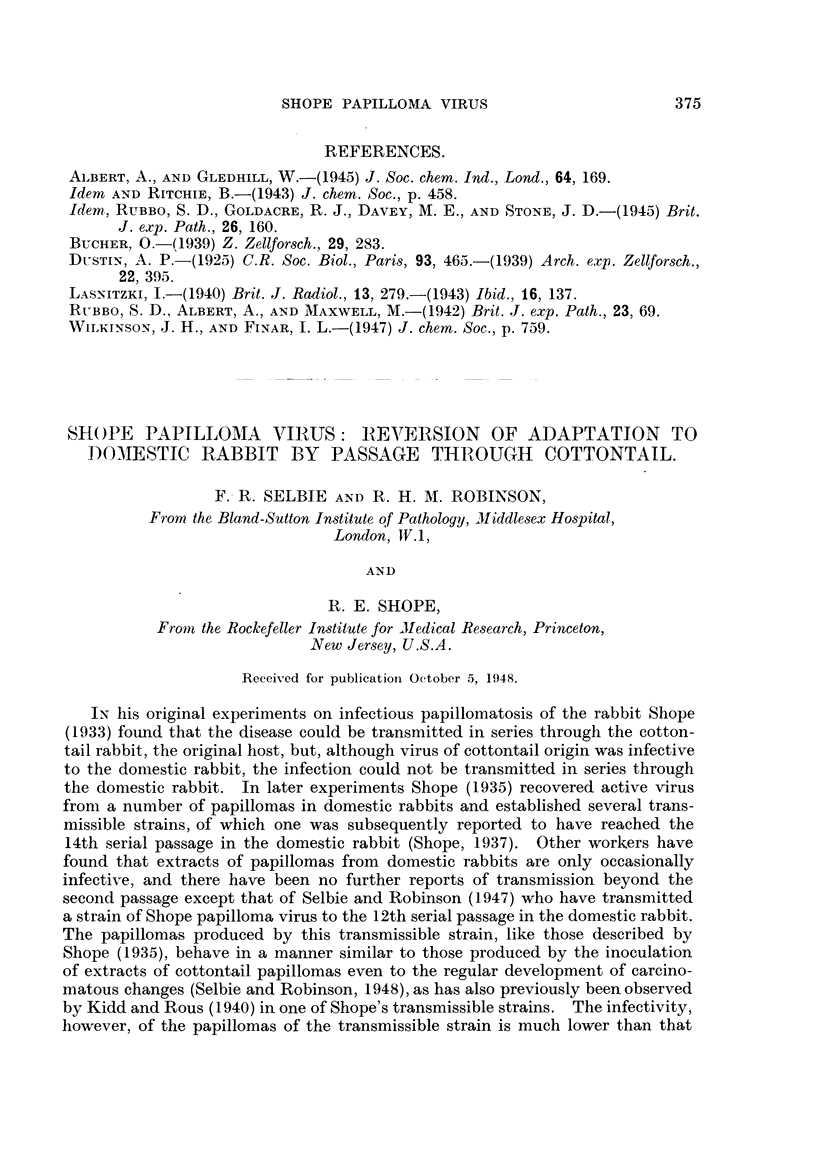# The Effect of Acridine Derivatives on Growth and Mitoses of Cells In Vitro

**DOI:** 10.1038/bjc.1948.40

**Published:** 1948-12

**Authors:** I. Lasnitzki, J. H. Wilkinson

## Abstract

**Images:**


### THE EFFECT OF ACRIDINE DERIVATIVES ON GROWTH AND MITOSIS OF CELLS *IN VITRO*.

I. LASNITZKI* AND J. H. WILKINSON.

*From the Strangeways Research Laboratory, Cambridge, and the Department of Chemical Pathology, Westminster Medical School (University of London), 17, Horseferry Road, London, S.W.1.*

Received for publication November 15, 1948.

In view of the recent developments in the chemotherapy of acridines it seemed desirable to extend the study of Dustin (1925) and Bucher (1939) on the antimitotic action of 2:8-diamino-10-methylacridinium chloride (trypaflavine) to a number of new acridine derivatives. The effect of the following compounds on cells growing *in vitro* has therefore been examined :

5-aminoacridine hydrochloride,
5-amino-10-methylacridinium bromide,
5-amino-1-phenylacridine hydrochloride,
5-amino-1-phenyl-10-methylacridinium bromide, and
2-chloro-5-amino-10-methylacridinium bromide.

Tissue cultures which are simple living units without a blood or nerve supply are ideal material on which to demonstrate the direct effect of chemical or physical agents upon the cell. Owing to the simplification and standardization of experimental conditions which the method provides it is also possible to obtain precise quantitative data of the effect.

### TECHNIQUE.

The cultures used for this work were obtained from the sclerotic of 11-day-old chick embryos, and grown by the hanging-drop technique in a medium consisting of equal parts of chick plasma and chick embryo extract. Two subcultivations were made before carrying out the experiment. Various concentrations of the acridine derivatives were then added to the medium of the experimental cultures, while the control cultures received an equal amount of Tyrode solution. The final concentrations of each compound in the culture media are listed in Tables I and II. For each concentration of the compounds six to eight experimental and six to eight control cultures were used.

After 24 hours' incubation, i.e. at the time when the number of cell divisions normally reaches a maximum, the experimental cultures showing growth and all the controls were fixed in Susa solution and stained with haematoxylin. All mitotic cells present in the zone of growth were counted in the treated and untreated cultures, and the results were expressed quantitatively as the percentage inhibition of mitosis in the treated cultures as compared with the controls (Table II). In cultures treated with 5-amino-1-phenyl-10-methylacridinium

* Sir Halley Stewart Research Fellow.

TABLE I.—*The Effect on Outgrowth of Aminoacridines in Treated Cultures.*

| | Concentration. | | | | | | | | | | | |
|---|---|---|---|---|---|---|---|---|---|---|---|---|
| | 1:30,000. | | 1:60,000. | | 1:120,000. | | 1:240,000. | | 1:480,000. | | 1:960,000. | |
| | Number of cultures in which outgrowth is— | | | | | | | | | | | |
| | Absent. | Present. | Absent. | Present. | Absent. | Present. | Absent. | Present. | Absent. | Present. | Absent. | Present. |
| 5-Aminoacridine hydrochloride  | 6 | 0 | 6 | 0 | 6 | 0 | 7 | 0 | 5 | 1 | 4 | 3 |
| 5-Amino-10-methyl-acridinium bromide  | 6 | 0 | 6 | 0 | 5 | 1 | 4 | 2 | | .. | | .. |
| 5-Amino-1-phenyl-acridine hydrochloride  | 6 | 0 | 6 | 1 | 2 | 5 | | .. | | .. | | .. |
| 5-Amino-1-phenyl-10-methylacridinium bromide  | 2 | 6 | 0 | 6 | 0 | 6 | | .. | | .. | | .. |
| 2-Chloro-5-amino-10-methylacridinium bromide  | 6 | 0 | 6 | 0 | 6 | 0 | 5 | 1 | 0 | 6 | | .. |

bromide at 1/30,000 and 1/60,000 the incidence of cell degeneration was determined by counting resting and degenerating cells in several comparable fields of experimental and control cultures. The result was expressed as a percentage of resting cells. The values shown in Table II represent the increase of degenerate cells in treated cultures as compared with the controls.

TABLE II.—*Showing Percentage Inhibition of Mitosis in Treated Cultures as Compared with Control Cultures.*

| | Concentration. | | | | | |
|---|---|---|---|---|---|---|
| | 1/30,000. | 1/60,000. | 1/120,000. | 1/240,000. | 1/480,000. | 1/960,000. |
| 5-Aminoacridine hydrochloride | 100 | 100 | 100 | 100 | 58 ± 8·5 | 51 ± 8·4 |
| 5-Amino-10-methylacridinium bromide | 100 | 100 | 100 | 75 ± 4·8 | .. | .. |
| 5-Amino-1-phenylacridine hydrochloride | 100 | 83 ± 1·5 | 61 ± 2·1 | .. | .. | .. |
| 5-Amino-1-phenyl-10-methylacridinium bromide | 93 ± 1·7 Increase of degenerate cells 10% ± 3·2 | 54 ± 4·5 Increase of degenerate cells 21% ± 4·5 | 0 ± 7·9 | .. | .. | .. |
| 2-chloro-5-amino-10-methylacridinium bromide | 100 | 100 | 100 | 100 | 3 ± 6·2 | .. |

RESULTS.

The growth of tissue cultures is due to two factors: cell migration and mitosis. In the type of culture used cells begin to wander out of the explant after a few hours' incubation: mitosis begins several hours later and reaches a maximum about the 24th hour, by which time a zone of new tissue consisting of resting, dividing cells and, as a rule, a few degenerate cells, has formed around the explant.

In the higher concentrations all five compounds caused a considerable disturbance of cell migration. In many cultures growth was entirely absent or only a few cells protruded from the explant, while in others the zone of growth was substantially reduced (Table I, Fig. 1–6).

All the five compounds tested had also a marked antimitotic action of varying degree. The greatest effect both on migration and mitosis was seen in cultures treated with 5-aminoacridine hydrochloride. There was no outgrowth at all at concentrations greater than 1:240,000. At 1:480,000 five of the six treated cultures showed no outgrowth, and in the sixth there was a mitotic inhibition of 58 per cent. The compound was still effective at a concentration of 1:960,000 ; only four of the seven treated cultures had normal outgrowth, and mitosis was reduced to half the normal value.

The introduction of a quaternary methyl group into the 5-amino-acridine molecule considerably reduced the growth inhibitory effect. Thus in four of the six cultures treated with 5-amino-10-methylacridinium bromide at a concentration of 1:240,000 no outgrowth was observed, and the remaining two showed an inhibition of 75 per cent of mitosis. The presence of the chlorine atom in 2-chloro-5-amino-10-methylacridinium bromide appeared to counterbalance the effect of the methyl group, for at the same concentration (1:240,000) five of the six experimental cultures lack a zone of growth and, moreover, cell divisions were absent in the sixth which did show migration.

The presence of a 1-phenyl group caused a much more pronounced diminution of the growth inhibitory effect, for 5-amino-1-phenylacridine hydrochloride proved less active at 1:120,000 than the parent compound at 1:960,000. A summation of the effects of the 1-phenyl and 10-methyl groups seemed to occur in the case of 5-amino-1-phenyl-10-methylacridinium bromide. The outgrowth was very little affected by this substance. At 1:30,000 only two of the eight treated cultures showed absence of growth, but in the remaining six mitosis was very scarce, with 93 per cent inhibition. At 1:60,000 all cultures had a zone of growth with mitosis reaching half the normal value, while at 1:120,000 both outgrowth and mitosis were normal (Tables I and II).

The incidence of abnormal mitosis was not significantly increased in the treated cultures. This suggests that once the cells entered division they were able to complete it successfully in contrast to cells treated with colchicine (Dustin, 1939).

Hyperchromatosis and pycnosis of the nuclei similar to the cell degeneration seen and described in cells *in vitro* after radiation (Lasnitzki, 1940) were present

---

EXPLANATION OF PLATES.

FIG. 1–6 show growing edge of cultures fixed at 24 hours' incubation. × 66.

FIG. 1.—Untreated control culture.

FIG. 2.—Culture treated with 5-amino-1-phenyl-10-methylacridinium bromide at 1/30,000.

FIG. 3.—Culture treated with 5-amino-1-phenylacridine hydrochloride at 1/120,000.

FIG. 4.—Culture treated with 5-amino-10-methylacridinium bromide at 1/240,000.

FIG. 5.—Culture treated with 2-chloro-5-amino-10-methylacridinium bromide at 1/240,000.

FIG. 6.—Culture treated with 5-aminoacridine hydrochloride at 1/480,000.

FIG. 7–10 show groups of fibroblasts in normal and treated cultures fixed at 24 hours' incubation. × 1000.

FIG. 7.—Normal fibroblasts.

FIG. 8.—Abnormal vacuolization in fibroblasts treated with 5-amino-1-phenyl-10-methylacridinium bromide at 1/30,000.

FIG. 9.—Shrinkage of nucleoli in cells treated with 2-chloro-5-amino-10-methylacridinium bromide at 1/240,000.

FIG. 10.—Vacuolization of nucleoli in cells treated with 5-aminoacridine hydrochloride at 1/480,000.

Lasnitzki and Wilkinson.

7 8

9 10

Lasnitzki and Wilkinson.

in the experimental cultures. Degenerate cell counts in cultures treated with 5-amino-1-phenyl-10-methylacridinium bromide at 1/30,000 and 1/60,000 showed a marked increase over that of the controls at both concentrations (Table II).

The resting cells of the treated cultures were narrower and more pointed than the normal fibroblasts of the controls, and they did not stain as deeply with haematoxylin. Abnormal vacuolation was frequently observed in the cytoplasm Slightly affected cells showed one distinct vacuole in the region of the centrosphere, while in others a number of vacuoles could be seen. These finally coalesced to form one large vacuole occupying the whole cytoplasm (Fig. 8). In many cells the nucleoli appeared severely damaged: they were shrunk, rounded and stained excessively with haematoxylin. Around the shrunken nucleoli vacuoles appeared. In other cells the nucleoli formed ring-like structures with a central vacuole (Fig. 9, 10).

### DISCUSSION.

The diminution of activity caused by the introduction of a 1-phenyl group into the 5-aminoacridine molecule recalls the work of Albert, Rubbo, Goldacre, Davey and Stone (1945), who observed that in the acridine series bacteriostatic activity is paralleled by the degree of ionization of the molecule at the pH of the medium. The presence of the electron-attracting phenyl group in close proximity to the ring nitrogen atom weakens the basic strength of the compound, a fact determined experimentally by these workers. Its percentage ionization at the biologically important pH is therefore correspondingly reduced. In accordance with their theory 5-amino-1-phenylacridine proved to have weaker antibacterial properties than 5-aminoacridine. It appears possible that some similar rule may apply to the antimitotic properties of the series, but confirmation of this suggestion must await the accumulation of additional data.

Rubbo, Albert and Maxwell (1942) have pointed out that at low concentrations the action of the acridine antiseptics is bacteriostatic rather than bactericidal, and the present work suggests that under such conditions this is probably due to the interference with cell division and not to a general toxic effect.

The observation that the incidence of abnormal mitosis was not significantly increased in the treated cultures indicates that the effect of the compounds tested is to *prevent* cells from *entering* mitosis rather than to interfere with the actual process of division.

The frequency and degree of the nucleolar changes go roughly parallel with the antimitotic activity of the compounds examined, and it is possible that the inhibition of cell division may be due to the disturbance of the nucleoli. On the other hand, the nucleolar damage may only accompany the other changes.

The increase in degenerate cells is considerably more marked in cultures treated with 5-amino-1-phenyl-10-methylacridinium bromide at 1:60,000, and showing less mitotic inhibition than in cultures which had received twice the amount of the compound. This finding suggests that the action of the acridines at the higher dilutions is not due to a direct toxic effect on the cells, but that the appearance of degenerate cells is linked with mitotic inhibition and due to a breakdown of premitotic cells attempting division, a sequence of events observed in cultures exposed to radiation (Lasnitzki, 1943).

### EXPERIMENTAL.

A commercial specimen of 5-aminoacridine hydrochloride was used in this work, and the samples of 5-amino-10-methylacridinium bromide (Albert and Ritchie, 1943) and its 2-chloro derivative (Wilkinson and Finar, 1947) were prepared by the literature methods.

5-*Amino*-1-*phenylacridine hydrochloride* was prepared from the corresponding base (Albert and Gledhill, 1945). It crystallized from water in sparingly soluble fine lemon-yellow prisms, m.p. over 300°. (Found: N, 8·8; Cl, 10·95. $C_{19}H_{14}N_2$, HCl, $H_2O$ requires N, 8·65; Cl, 10·95 per cent.)

5-*Amino*-1-*phenyl*-10-*methylacridinium bromide.*—5-Amino-1-phenyl-acridine (1 g.) was heated at 105° for 30 minutes with acetic anhydride (4 c.c.) and the cooled mixture was poured into benzene (20 c.c.). The precipitated 5-acetamido-1-phenylacridine (0·97 g.) was collected by filtration and crystallized from alcohol or pyridine. It separated in pale yellow needles, m.p. 284°. The acetyl derivative (0·5 g.) was dissolved in nitrobenzene (5 c.c.), and heated with dimethyl sulphate (0·7 g.) at 140° for 30 minutes. The cooled mixture was poured into benzene (30 c.c.) and set aside overnight. The gummy product was separated by decantation and hydrolysed by heating with 48 per cent hydrobromic acid (5 c.c.) on a steam bath for one hour. The required 5-*amino*-1-*phenyl*-10-*methylacridinium bromide* which separated on cooling crystallized from water in yellow prisms, m.p. over 300°. (Found: N, 7·65. $C_{20}H_{17}N_2Br$ requires N, 7·7 per cent.) An aqueous solution gave no precipitate with N sodium bicarbonate, but sodium hydroxide gave a yellow precipitate of the corresponding *pseudo* base.

### SUMMARY.

Chick fibroblasts were grown in a medium containing a number of new acridine derivatives and their effect on outgrowth and mitotic rate was examined.

The following compounds which were added to the culture medium in concentrations ranging from 1:30,000 to 1:960,000 were used:

5-aminoacridine hydrochloride,
5-amino-10-methylacridinium bromide,
5-amino-1-phenylacridine hydrochloride,
5-amino-1-phenyl-10-methylacridinium bromide,
2-chloro-5-amino-10-methylacridinium bromide.

All the substances caused disturbance of outgrowth and/or reduction of mitosis of varying degree. 5-aminoacridine hydrochloride proved to have the greatest and 5-amino-1-phenyl-10-methyl-acridinium bromide the smallest inhibitory effect.

At the higher concentrations rounding and vacuolization of the nucleoli and vacuolization of the cytoplasm could be observed. The effect of the compounds on mitosis was found to resemble that of small doses of radiation: cells were prevented from entering division, but there was no interference with the actual process of division in contrast to colchicine.

The bearing of the degree of ionization of the substances used on their growth inhibitory action is discussed.

The authors wish to thank Mr. G. Lenney for the photomicrographs.

### REFERENCES.

ALBERT, A., AND GLEDHILL, W.—(1945) *J. Soc. chem. Ind., Lond.*, **64**, 169.
*Idem* AND RITCHIE, B.—(1943) *J. chem. Soc.*, p. 458.
*Idem*, RUBBO, S. D., GOLDACRE, R. J., DAVEY, M. E., AND STONE, J. D.—(1945) *Brit. J. exp. Path.*, **26**, 160.
BUCHER, O.—(1939) *Z. Zellforsch.*, **29**, 283.
DUSTIN, A. P.—(1925) *C.R. Soc. Biol., Paris*, **93**, 465.—(1939) *Arch. exp. Zellforsch.*, **22**, 395.
LASNITZKI, I.—(1940) *Brit. J. Radiol.*, **13**, 279.—(1943) *Ibid.*, **16**, 137.
RUBBO, S. D., ALBERT, A., AND MAXWELL, M.—(1942) *Brit. J. exp. Path.*, **23**, 69.
WILKINSON, J. H., AND FINAR, I. L.—(1947) *J. chem. Soc.*, p. 759.